# Dr. Philip, on the Action of the Heart

**Published:** 1817-07

**Authors:** C. P. Wilson Philip


*To the Editors of the Medico-Chirurgical Journal & Review.*

GENTLEMEN, Worcester, June 3, 1817.

In the last Number of the Medico-Chirurgical Journal, I am called upon to explain an apparent contradiction in some observations in a paper of mine on the action of the heart, and the results of Dr. Parry's experiments, in his work on the arterial pulse. If the reader particularly compares Dr. Parry's experiments and mine, the contradiction, which strikes him at first view, will, I think, disappear. By the beatings of the arteries, I mean the motions observed in them corresponding to the beatings of the heart. Whether these motions are occasioned by alternate dilatation and contraction of the arteries, I did not inquire. In most of Dr. Parry's experiments, made for the purpose of examining the motions of the arteries, he observed a motion in them corresponding to that of the heart. This was the case in Experiments 6, 8, 9, 10, 11, 12, 13, 14, 15, 16. Most of the experiments which follow these, were made for the purpose of observing the effects of ligatures thrown round the arteries. After this was done, a motion in the upper part of the vessel, from the beating of the heart, could generally be perceived. Of the horse he says (Experiment 21), "In this, as in other experiments, when the neck was considerably bent, the carotid, where it was separated from its attachments, rose in a curve, outwards, at each systole of the ventricle."

We might, a priori, suppose, that in experiments of this kind the artery will sometimes be little, if at all, sensibly moved by the action of the heart, which may often be much weakened both by loss of blood and the effects of fear. The impression on my mind is, that in rabbits I have almost always perceived some motion in the arteries corresponding to the beating of the heart, when the circulation was pretty vigorous. In frogs my experiments were made on the heart and capillaries, and I do not recollect observing or mentioning the state of the larger arteries in them, which however, I should suppose, will be found much the same as in rabbits, the circulation in the former appearing to be equally vigorous. My experiments were generally made immediately after sensible death, the circulation being maintained by artificial respiration. It is probable that, under these circumstances, as no feeling of the animal interferes, the motion of the arteries will be most uniformly observed. It may often be seen in the human body while the skin is entire. I have often seen it both in the temple and wrist; and have known more than one person, in whom the beating of the carotids was so strong, particularly after dinner, that the pulse could easily be counted by observing the motions of their neckcloths. I shall soon have occasion to lay before the public some observations on the inferences from Dr. Parry's very accurate experiments. You will oblige me by giving a place to the above explanation in the next Number of the Medico-Chirurgical Journal.

I have the honour to be, Gentlemen,

Your obedient humble servant,

C. P. WILSON PHILIP.

